# Metabolic Syndrome and Neurocognitive Function among older Hispanics/Latinos with HIV

**DOI:** 10.1093/geroni/igab046.2514

**Published:** 2021-12-17

**Authors:** Maria Marquine, Lily Kamalyan, David Yassai-Gonzalez, Mariana Cherner, Ronald Ellis, Anya Umlauf, Dilip Jeste, Robert Heaton

**Affiliations:** 1 University of California San Diego, La Jolla, California, United States; 2 San Diego State University/University of California San Diego Joint Doctoral Program in Clinical Psychology, San Diego, California, United States; 3 University of California San Diego, SAN DIEGO, California, United States; 4 University of California San Diego, San Diego, California, United States

## Abstract

Neurocognitive impairment is prevalent among persons with HIV (PWH), particularly among Hispanics/Latinos/as/x (henceforth Hispanics). We examined disparities in HIV-associated neurocognitive function between older Hispanic and non-Hispanic White PWH, and the potential role of metabolic syndrome (MetS) in explaining these disparities. Participants included 116 community-dwelling PWH ages 50-75, who were enrolled in a cohort study in southern California (58 Hispanic [53% Spanish-speaking] and 58 age-comparable non-Hispanic White; Overall group: Age: M=57.9, SD=5.7; Education: M=13, SD=3.4; 83% male, 58% AIDS, 94% on antiretroviral therapy [ART], 4% detectable plasma RNA). A global neurocognition score was derived from T-Scores on a comprehensive neurocognitive battery, with separate demographic adjustments for English and Spanish-speakers. MetS was ascertained via standard criteria that considered central obesity, elevated triglycerides, low high-density lipoprotein cholesterol, and elevated fasting glucose, as well as current medical treatment for these conditions. Covariates examined included sociodemographic, psychiatric, substance use and HIV-disease characteristics. Hispanics had higher rates of MetS (56%) than non-Hispanic Whites (37%; p<.05). A stepwise regression model on global neurocognition including ethnicity and covariates that differed between ethnic groups, selected only Hispanic ethnicity as a significant predictor (B=-3.82, SE=1.27, p<.01). A comparable model also including MetS showed that both Hispanic ethnicity (B=-3.39, SE=1.31, p=.01) and MetS (B=-2.73, SE=1.31, p=.04), were significantly associated with worse global neurocognition. Findings indicate that MetS does not fully explain disparities in neurocognitive function between Hispanic and non-Hispanic White older PWH, but rather is an independent predictor of neurocognitive function along with Hispanic ethnicity.

